# Simulating treatment effects for gonorrhoea using a within-host mathematical model

**DOI:** 10.1016/j.idm.2026.01.002

**Published:** 2026-01-27

**Authors:** Pavithra Jayasundara, David G. Regan, Philip Kuchel, James G. Wood

**Affiliations:** a School of Population Health, UNSW Sydney, NSW, Australia; b The Kirby Institute, UNSW Sydney, NSW, Australia; c School of Life and Environmental Sciences, University of Sydney, NSW, Australia

**Keywords:** Gonorrhoea, Pharmacokinetic, Pharmacodynamic, Gepotidacin, Intracellular, Gentamicin, Azithromycin

## Abstract

*Neisseria gonorrhoeae* (NG) bacteria have evolved resistance to many of the antibiotics used to treat gonorrhoea infection. To explore potential treatment options for gonorrhoea, we extend a previously developed within-host mathematical model to integrate treatment dynamics by accounting for key pharmacokinetic (PK) and pharmacodynamic (PD) features. This extended model was used to investigate different treatment regimens for two potential drugs: monotreatment with gepotidacin, and dual treatment with gentamicin and azithromycin. The simulated treatment success rates aligned well with the limited clinical trial data available. The simulation results indicated that antibiotic treatment failure is associated with failure to successfully clear intracellular NG (NG residing within epithelial cells and neutrophils), and extracellular PK indices alone cannot differentiate between treatment success/failure. Also, the index defined by the ratio of area under the curve to minimum inhibitory concentration (AUC/MIC) index >150, evaluated using intracellular gepotidacin concentration, successfully distinguished between treatment success and failure. For the dual treatment regimen, AUC/MIC index >140 evaluated using the simulated single drug concentration, representing the combined effect of gentamicin and azithromycin with the Loewe additivity concept, successfully differentiated between treatment success and failure. However, we found this PK threshold associated with dual treatment to be less informative than that of gepotidacin, as a majority of samples below this threshold still resulted in infection clearance. Although previous experimental results on antibiotic killing of intracellular NG are scarce, our findings highlight the need for further studies on this. This will be useful for testing putative new anti-gonorrhoea antibiotics.

## 1 Introduction

Gonorrhoea is a sexually transmitted infection caused by bacteria of the species *Neisseria gonorrhoeae* (NG). Since the beginning of the antibiotic era, NG has progressively developed resistance to the classes of drugs used to treat gonorrhoea, and current treatments are now under threat with few alternatives of proven safety and efficacy (Terreni et al., 2021; Unemo et al., 2016). Drug resistant NG has become a major public health concern (Centers for Disease Control and Prevention, 2019; World Health Organization, 2012) and the development of new treatment options and prophylactic vaccines is seen as increasingly important in population control of gonorrhoea. In many low and middle-income countries (LMIC), ceftriaxone and azithromycin dual therapy remains the predominant treatment despite rising global resistance. Reports of multidrug-resistant NG with reduced susceptibility to ceftriaxone, such as recent isolates described in Thailand (Kueakulpattana et al., 2021), highlight the growing fragility of existing treatment strategies and the urgent need for alternative therapeutic options.

In clinical trial settings both gepotidacin (GEP) (Scangarella-Oman et al., 2018; Taylor et al., 2018) and gentamicin (GEN) + azithromycin (AZM) dual treatment (Kirkcaldy et al., 2014; Rob et al., 2020; Ross et al., 2019) have shown potential for treating urethral NG infection. Gepotidacin is a novel triazaacenaphthylene bacterial type II topoisomerase inhibitor while azithromycin is a macrolide and gentamicin is an aminoglycoside. Both macrolides and aminoglycosides work by disrupting bacterial protein synthesis by inhibiting ribosome functionality (Brooks et al., 2013). Clinical trials report much higher treatment effectiveness using dual therapy with gentamicin + azithromycin (100 % cure rate (Kirkcaldy et al., 2014)) than with gentamicin monotherapy (68–98 % cure rate (Dowell & Kirkcaldy, 2013)), but similar effectiveness to using azithromycin monotherapy (99.2 % cure rate (Moran & Levine, 1995)). By comparing the minimum inhibitory concentration (MIC) of azithromycin and gentamicin on NG strains under monotherapy and dual therapy, the *in vitro* study by Xu et al. (2022) has shown that when used in combination, gentamicin can decrease the progression of the development of azithromycin resistance. This combination therapy is recommended as an alternative treatment for patients who cannot be treated with the recommended treatment ceftriaxone, due to infection with ceftriaxone resistant strains (Xu et al., 2022), allergy or unavailability of ceftriaxone.

Although clinical trials are considered the gold standard for evaluating the safety and effectiveness of new drugs, they have limitations in terms of expense, duration and ethical constraints, which compromise their utility for optimising doses, regimens and drug combinations (Hook et al., 2020). In this case, simulations through compartment pharmacokinetic (PK)/pharmacodynamic (PD) models such as those used in the study by Chisholm et al. (2010) are useful in determining effective dosing regimens. In the context of NG, a within-host mechanistic model has the potential to explore intracellular treatment effects for which there is little experimental evidence. In our previous work on within-host modelling of natural NG infection (Jayasundara et al., 2019), we observed that intracellular survival and replication of NG appears to be a key factor in prolonging untreated infection. Therefore, it is of interest to consider how treatment resolves infection while accounting for intracellular NG states.

In this context intracellular PK/PD effects appear likely to be essential in guiding the design of treatment regimens. However, while experimental studies of extracellular PK/PD effects for NG infection (e.g. (Connolly et al., 2019; VanScoy et al., 2020), have been conducted, we were unable to find any studies that explored intracellular PK/PD effects in the context of NG infection.

The primary objective of this study is to extend an existing within-host mathematical model of male urethral NG infection (Jayasundara et al., 2019) to incorporate antibiotic pharmacokinetic and pharmacodynamic effects, with a particular focus on how intracellular NG dynamics influence treatment outcomes. As secondary aims, we investigate the role of intracellular NG in determining MIC for treatments evaluated in recent trials as future options: monotreatment with gepotidacin (GEP) and dual treatment with gentamicin (GEN) + azithromycin (AZM); and also analyse extracellular and intracellular PK/PD interactions to determine drug concentration levels required for bacteriological clearance across these treatment strategies. In our analysis of gepotidacin, it is important to note that this drug is not yet widely available, particularly in LMIC where gonorrhoea prevalence is high. Therefore, our evaluation of gepotidacin should be viewed as a forward-looking assessment of a potential future treatment option rather than a reflection of currently available therapies.

## 2 Materials and methods

### 2.1 Mathematical model of antibiotic treatment

In Jayasundara et al. (2019), we developed a deterministic compartmental within-host transmission model to describe untreated symptomatic male urethral infection with NG which was compared and validated against available data from human experimental models to understand the long term dynamics of untreated infection. In that model, four NG states (unattached NG (*B*), NG attached to epithelial cells ($B_{a}$), NG internalised within epithelial cells ($B_{i}$) and NG surviving within polymorphonuclear leukocytes (PMN) ($B_{s}$)) and the innate immune response mediated by PMN are used to describe the infection process. In this study, we extend this model to include treatment effects by applying PK/PD principles. The use of *in vitro* time-kill data (Farrell et al., 2017; Foerster et al., 2016) allows us to parameterise NG killing at different antibiotic concentrations (PD), which when paired with relevant PK parameters, allows us to model the antibiotic concentration in-host and conduct longer-term projections of the bacterial populations using the killing relationship established via the time-kill data.

In this study, we incorporate treatment effects in both extracellular (*B* and $B_{a}$) and intracellular NG states ($B_{i\;}$ and $B_{s}$) using drug-specific Hill functions (Foerster et al., 2016), with differing concentrations of drug in the extracellular and intracellular environments. The Hill function parameters are estimated using the NG growth data reported in the *in vitro* time-kill experiments for gentamicin and azithromycin conducted by Foerster et al. (2016) and, for gepotidacin, in the study by Farrell et al. (2017). We use the *in vitro* time-kill experiments to determine the growth rate of NG under treatment for a given antibiotic as a function of drug concentration, assumed to follow a Hill function as defined in Eq. (1). The Hill function is determined by four parameters: the maximum ($\varphi_{\max}$) and minimum ($\varphi_{\min}$) bacterial growth rates in the absence and presence of the antibiotic, respectively; the *zMIC* which is the pharmacodynamic MIC as referred in Regoes et al. (2004); and the Hill coefficient ($k_{H}$), which reflects the sensitivity of the change in the net bacterial growth rate to the changes in the antibiotic concentration φ(C). Under this parameterisation, φ(C) is then described by Eq. (1):

(1) $$\varphi \left(C\right)=\varphi_{\max}-\frac{\left(\varphi_{\max}-\varphi_{\min}\right){\left(\frac{C}{zMIC}\right)}^{k_{H}}}{{\left(\frac{C}{zMIC}\right)}^{k_{H}}-\frac{\varphi_{\min}}{\varphi_{\max}}}$$

Using the *in vitro* time kill studies (Farrell et al., 2017; Foerster et al., 2016) we were able to estimate the Hill function parameters by regressing the NG load observations against time as in Foerster et al. (2016), NG growth is measured hourly for 6 h (0, 1, 2, 3, 4, 5, 6 h time points) and in the study Farrell et al. (2017) it is measured at 0, 2, 4, 8 and 24 h time points. Further details are described in Appendix S1 Section S1.

**Fig. 1:**
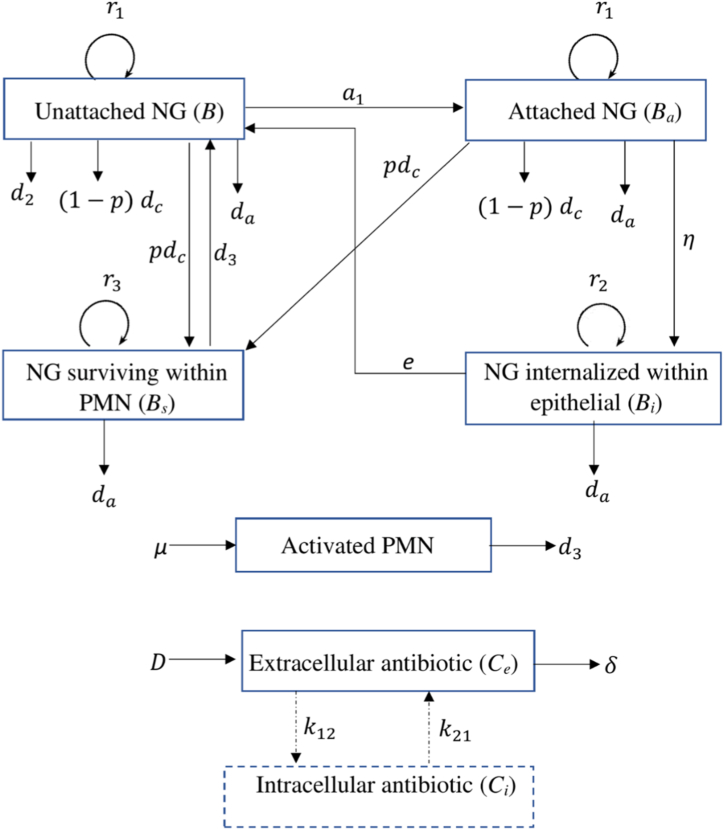
Schematic illustration of the within-host NG infection model including antibiotic treatment. Arrows indicate transitions between model states (boxes). Antibiotic- and PMN-mediated killing of NG are denoted as $d_{a}$ and $d_{c}$, respectively (for killing by PMN see Jayasundara et al. (2019)). The replication rates of NG in different states are denoted as $r_{1}$ – replication rate of non-internalised NG (unattached NG and NG attached to epithelial cells), $r_{2}$ – replication rate of NG internalised within epithelial cells and $r_{3}$ – replication rate of NG surviving within PMN. The parameter values of these replication rates are described in Jayasundara et al. (2019). Explicit intracellular antibiotic compartments are included for gentamicin and azithromycin (see Section ‘Mathematical model of antibiotic treatment’), with transitions between extra and intracellular drug concentrations (dashed lines) applying only for these two drugs.

The dual treatment effects of gentamicin and azithromycin are modelled using the concept of Loewe additivity. This approach is appropriate when drugs share similar mechanisms of action and targets that one drug can, in principle, replace some proportion of the other. Under this assumption, the combined effect of the drugs can be expressed in terms of an equivalent concentration of either drug alone. Gentamicin and azithromycin both inhibit bacterial protein synthesis by binding to the ribosomal complex, and previous modelling studies have successfully applied Loewe additivity to aminoglycoside–macrolide combinations (Mensforth & Ross, 2019; Ross et al., 2019). When modelling gepotidacin concentration, we adopt a one-compartment model (Rowland & Tozer, 1995) as has been applied by So et al. (2015) where we assume that drug concentration declines exponentially on a time-scale determined by the half-life of the drug. However, for gentamicin (Tulkens, 1991; Tulkens & Trouet, 1978) and azithromycin (Foulds et al., 1990; Wildfeuer et al., 1996), we adopt a two-compartment model to account for more complex intracellular drug distribution and accumulation. Model specific parameter values are given in Table 1, with the treatment model described in greater detail in Appendix S1 and the parameters describing untreated infection described in detail in Jayasundara et al. (2019). Fig. 1 provides a schematic illustration of the natural infection model with the added treatment effects.

**Table 1:** Model parameter values for the three antibiotics considered in this study: gepotidacin (GEP), gentamicin (GEN) and azithromycin (AZM).

Symbol	Parameter (units)	Drug	Point Estimate (LHS range)	References/Comments
*D*	Initial antibiotic dose (mg)	GEP	1500/3000	Trial doses (Scangarella-Oman et al., 2018; Taylor et al., 2018).
		GEN	240	Trial doses (Brittain et al., 2016; Hira et al., 1985).
		AZM	1000	CDC recommended dose for dual treatment (Centers for Disease Control and Prevention, 2015).
$b_{a}$	Bioavailability	GEP	0.44 (0.38–0.5)	(Negash et al., 2016; Tiffany et al., 2014)
		GEN	1	Given intramuscularly (Katzung, 2018).
		AZM	0.37	Foulds et al. (1990)
$V_{d}$	Volume of distribution (L)	GEP	188.7	Negash et al. (2016)
		GEN	16.8 (10–20)	Al-Lanqawi et al. (2009)
		AZM	3219 (1593–5475)	Ripa et al. (1996)
$f_{u}$	Fraction unbound	GEN	0.85–1	Bailey and Briggs (2004)
		AZM	0.88	Singlas (1995)
		GEP	0.76	Bulik et al. (2017)
α	The ratio of intracellular to extracellular drug concentration	GEP	1.8 (1.5–2.5)	Peyrusson et al. (2018)
$C_{e}\left(0\right)$	Initial extracellular drug concentration level (mg/L)	GEP	2.64 (2.43–3.04)	Computed using the formula $\frac{D\times b_{d}\times f_{u}}{V_{d}}$ (Austin et al., 1998)
		GEN	14.29 (10.21–23.76)	
		AZM	10.85 (9.31–13.02)	
$C_{i}\left(0\right)$	Initial intracellular drug concentration level (mg/L).	GEP	4.75 (3.65–7.6)	Computed using the formula α*×*$C_{e}\left(0\right)$ (Chisholm et al., 2010)
		GEN	0	Drug enters from the extracellular compartment.
		AZM	0	
$k_{12}$	Transfer rate constant from the extracellular to intracellular compartment (h^−1^)	GEN	0.04 (0.03–0.04)	Point estimate from Schentag et al. (1977), range refined via calibration with susceptibility breakpoint. Point estimate from Ripa et al. (1996), range refined via calibration with susceptibility breakpoint.
		AZM	0.12 (0.10–0.18)	
$k_{21}$	Transfer rate constant from the intracellular to extracellular compartment (h^−1^)	GEN	0.01 (0.008–0.016)	Schentag et al. (1977)
		AZM	0.04 (0.03–0.06)	Point estimate from Ripa et al. (1996), range refined via calibration with susceptibility breakpoint.
$V_{e}$	Volume of the extracellular compartment (L)	AZM	569 (485–779)	Point estimate from Ripa et al. (1996), range refined via calibration with susceptibility breakpoint.
		GEN	0.95 (0.60–1.29)	Point estimate from Schentag et al. (1977), range refined via calibration with susceptibility breakpoint.
$V_{i}$	Volume of the intracellular compartment (L)	AZM	1779 (981–1916)	Point estimate from Ripa et al. (1996), range refined via calibration with susceptibility breakpoint.
		GEN	0.23 (0.18–0.27)	Schentag et al. (1977)
δ	Rate constant of drug elimination (h^−1^)	GEP	0.06 (0.05–0.07)	Point estimate as $\frac{\log\left(2\right)}{half-life}$ using Negash et al. (Negash et al., 2016). The lower and upper limit of the LHS ranges are based on Hossain et al. (2014) and Tiffany et al. (2014) respectively.
		GEN	0.14 (0.11–0.18)	Elimination rate constant in Schentag et al. (1977).
		AZM	0.08 (0.05–0.10)	Elimination rate constant in Ripa et al. (1996).
$\varphi_{\min}$	Minimum bacterial growth rate constant in the presence of antibiotic $\left(h^{-1}\right)$	GEP	−0.53 (−0.64, −0.46)	Estimated by fitting to data in Farrell et al. (2017).
		GEN	−8.18 (−10.00, −6.35)	Estimated by fitting to data in Foerster et al. (2016).
		AZM	−1.50 (−2.06, −0.99)	
$k_{H}$	The Hill coefficient	GEP	2.47 (1.78, 3.64)	Estimated by fitting to data in Farrell et al. (2017).
		GEN	1.70 (1.14, 2.64)	Estimated by fitting to data in Foerster et al. (2016).
		AZM	0.91 (0.70, 1.32)	
$\varphi_{\max}$	Maximum bacterial growth rate constant in the absence of antibiotic $\left(h^{-1}\right)$	GEP	0.79 (0.76–0.84)	Estimated by fitting to data in Farrell et al. (2017).
		GEN	0.89 (0.82–0.91)	Estimated by fitting to data in Foerster et al. (2016).
		AZM	0.63 (0.61–0.69)	
*zMIC*	Minimum inhibitory concentration (mg/L)	GEP	0.26 (0.20, 0.32)	Estimated by fitting to data in Farrell et al. (2017).
		GEN	0.24 (0.17, 0.32)	Estimated by fitting to data in Foerster et al. (2016).
		AZM	0.03 (0.02, 0.33)

**Table 2:** Susceptibility breakpoints (mg/L) derived from the three sub-models and the full model and comparison with empirical breakpoints.

Drug	Susceptibility breakpoints (mg/L)				
	Model A	Model B	Model C	Full model point estimate (LHS range)	Empirical breakpoints
GEP	2.55	0.79	0.73	0.64 (0.48–1.1)	Not available
AZM	9.35	0.89	0.70	0.69 (0.55–1.29)	0.5 (European Committee on Antimicrobial Susceptibility Testing, 2018), 1 (Clinical and Laboratory Standards Institute, 2017)
GEN	12.75	1.94	1.74	1.60 (1.51–5.54)	4 (Brown et al., 2010).

The model equations for the four NG states under treatment are given below, with treatment incorporated using the pharmacokinetic models described above.

$$\frac{dB}{\text{dt}}=\left(1-\frac{B+B_{a}}{k_{1}}\right)\left(r_{1}\;B+d_{3}\;B_{s}+e{\;B}_{i}\right)-d\frac{B\;N}{c\;N+B}-d_{2}\;B-{\;a}_{1}\;B\left(1-\frac{B_{a}}{k_{1}\;a_{2}}\right)-\frac{\left(r_{1}-\varphi_{\min}\right){\left(\frac{C_{e}}{MIC}\right)}^{k_{H}}}{{\left(\frac{C_{e}}{MIC}\right)}^{k_{H}}-\frac{\varphi_{\min}}{r_{1}}}\;B\left(1-\frac{B+B_{a}}{k_{1}}\right)$$

$$\frac{dB_{a}}{\text{dt}}=r_{1}{\;B}_{a}\left(1-\frac{B+B_{a}}{k_{1}}\right)+{\;a}_{1}\;B\left(1-\frac{B_{a}}{k_{1}\;a_{2}}\right)-d\frac{B_{a}\;N}{c\;N+B_{a}}-\eta \;B_{a}-\frac{\left(r_{1}-\varphi_{\min}\right){\left(\frac{C_{e}}{MIC}\right)}^{k_{H}}}{{\left(\frac{C_{e}}{MIC}\right)}^{k_{H}}-\frac{\varphi_{\min}}{r_{1}}}\;B_{a}\left(1-\frac{B+B_{a}}{k_{1}}\right)$$

$$\frac{dB_{i}}{\text{dt}}=\left(1-\frac{B_{i}}{k_{1}{\;a}_{2}}\right)\left(\eta {\;B}_{a}+r_{2}{\;B}_{i}\right)-e{\;B}_{i}-\frac{\left(r_{2}-\varphi_{\min}\right){\left(\frac{C_{i}}{MIC}\right)}^{k_{H}}}{{\left(\frac{C_{i}}{MIC}\right)}^{k_{H}}-\frac{\varphi_{\min}}{r_{2}}}\;{\;B}_{i}\left(1-\frac{B_{i}}{k_{1}{\;a}_{2}}\right)$$

$$\frac{dB_{s}}{\text{dt}}=\left(1-\frac{B_{s}}{{N\;k}_{2}}\right)\left(p\;d\frac{B\;N}{c\;N+B}+p\;d\frac{B_{a}\;N}{c\;N+B_{a}}+r_{3}\;B_{s}\right)-d_{3\;}B_{s}-\frac{\left(r_{3}-\varphi_{\min}\right){\left(\frac{C_{i}}{MIC}\right)}^{k_{H}}}{{\left(\frac{C_{i}}{MIC}\right)}^{k_{H}}-\frac{\varphi_{\min}}{r_{3}}}\;B_{s}\;\left(1-\frac{B_{s}}{{N\;k}_{2}}\right)$$

$$\frac{dN}{\text{dt}}=\mu \;\left(N_{\max}-N\right)\;\left(B+B_{a}\right)-d_{3}\;N$$

For gepotidacin (one-compartment model):

$$\frac{dC_{e}\;}{dt}=-\;\delta C_{e}$$

$$C_{i}\left(t\right)=\alpha C_{e}\left(t\right)$$

For azithromycin and gentamicin (two-compartment model):

$$\frac{dC_{e}\;}{dt}=-\;\delta C_{e}-k_{12\;}C_{e}+k_{21\;}C_{i}\frac{V_{i}}{V_{e}}$$

$$\frac{dC_{i}}{dt}=k_{12\;}C_{e}\frac{V_{e}}{V_{i}}-k_{21\;}C_{i}$$

### 2.2 Incorporation of parametric uncertainty

To account for parametric uncertainty across the natural infection model, in (Jayasundara et al., 2019) we selected 5402 parameter sets, generated using Latin hypercube sampling (LHS), which met the relevant outcome criteria for the natural time-course of infection (here we index these LHS parameter sets as i=1,2,…,5402). To incorporate parameter uncertainty that is related to treatment, we extend this previous analysis by also simulating from the ranges that are associated with the treatment parameters. We achieve this by first generating 5402 uniform LHS samples (indexed as j=1,2,…,5402) for the PK/PD parameters using the parameter ranges derived from relevant literature and summarised in Table 1 and Appendix S1, Table S3. Then to incorporate both natural infection and treatment-related parametric uncertainty, the LHS parameter sets that satisfy the indexing i=j are combined to result in 5402 sets of parameter values. Using these 5402 samples, we assess the modelled infection clearance times.

### 2.3 Calibrating PK/PD parameters using susceptibility breakpoints

Explicitly capturing the development of antibiotic resistance would require considerable model extension with very limited data availability. Therefore, in this study, rather than directly modelling processes relating to antibiotic resistance, we vary the *zMIC* parameter in Eq. (1) as a proxy for changes in the susceptibility to a given treatment (Kjellander & Finland, 1963; Martin et al., 1970). The *in-vitro* time-kill experiments, described in the above section measure bacterial growth in the presence of antibiotics, using wild-type NG strains that do not express resistance against the tested antibiotics. To capture the notion of decreased susceptibility (or increased resistance) to treatment, we explore the effect of treatment via the *zMIC* parameter in the Hill function, which we increase gradually from the antibiotic-specific *zMIC* values estimated as described in Section ‘Mathematical model of antibiotic treatment’ for a susceptible NG strain. To this end, we determine a ‘model-derived susceptibility breakpoint’ such that for *zMIC* below and above the breakpoint, the infection clears in ≤7 days and >7 days, respectively (infection clearance threshold, as described in Section ‘Simulated treatment strategies’). In our simulation study, as we had to mostly rely on *in vitro* studies when estimating the model parameters, we then calibrate these model-derived breakpoints to reproduce the empirical breakpoints. Thereby we refine the ranges of the parameters that are influential in determining the model-derived susceptibility breakpoints with calibration details provided in Appendix S1, Section S3. Here, we define ‘empirical breakpoints’ as the relevant susceptibility breakpoints for azithromycin published by the Clinical and Laboratory Standards Institute (CLSI) (1 mg/L (Clinical and Laboratory Standards Institute, 2017)) and the European Committee on Antimicrobial Susceptibility Testing (EUCAST) (0.5 mg/L (European Committee on Antimicrobial Susceptibility Testing, 2018)). For gentamicin, a susceptibility breakpoint of 4 mg/L is defined based on epidemiological and clinical observations in Malawi as reported in the study by Brown et al. (2010). Furthermore, Brown et al. (2010) defines intermediate susceptibility for gentamicin for MIC 8–16 mg/L and resistance for MIC ≥32 mg/L. For gepotidacin which is not currently used in clinical practice, we use the breakpoints determined in the clinical trials conducted by Taylor et al. (2018) and Scangarella-Oman et al. (2018).

### 2.4 Simulated treatment strategies

In this study, we simulate the effectiveness of the single and multiple dose treatment strategies summarised in Table 3, Table 4. Here, we consider strategies that have been previously tested in clinical trials and compare the simulated treatment effectiveness with clinical trial results as well as using the model to simulate the effectiveness of several novel multiple-dose strategies. Therefore, as previously clinically tested strategies, for gepotidacin we analyse the effectiveness of 1500 mg and 300 mg single dose strategies (Scangarella-Oman et al., 2018; Taylor et al., 2018). For the dual treatment combination we test 240 mg GEN + 1 g AZM strategy tested in the clinical trial Kirkcaldy et al. (2014) and 240 mg GEN + 2 g AZM strategy tested in Rob et al. (2020).

**Table 3:** Percentage of simulations using LHS samples (out of 5402) that clear infection in ≤7 days when using single and multiple dose gepotidacin treatment strategies.

Treatment strategy	Percentage of simulations that clear infection				
	*zMIC* (mg/L)				
	0.05	0.125	0.25	0.5	1
1500 mg single dose	100.0	100.0	99.9	95.0	20.8
500 mg × 3, 8 h apart	100.0	100.0	100.0	99.2	38.1
500 mg × 3, 12 h apart	100.0	100.0	100.0	99.6	40.4
500 mg × 3, 24 h apart	100.0	100.0	100.0	98.9	14.0
3000 mg single dose	100.0	100.0	100.0	99.9	95.0
500 mg × 6, 8 h apart	100.0	100.0	100.0	100.0	99.8
500 mg × 6, 12 h apart	100.0	100.0	100.0	100.0	99.5
500 mg × 6, 24 h apart	100.0	100.0	100.0	100.0	66.2
1500 mg × 2, 8 h apart	100.0	100.0	100.0	100.0	95.7
1500 mg × 2, 12 h apart	100.0	100.0	100.0	100.0	98.4
1500 mg × 2, 24 h apart	100.0	100.0	100.0	100.0	99.3

**Table 4:** Percentage of simulations that clear the infection (out of 5402 LHS samples) at various *zMIC* values with gentamicin (GEN) and azithromycin (AZM) dual therapy regimens.

Treatment strategy	Percentage of simulations that clear infection					
	(Gentamicin/azithromycin) *zMIC* (mg/L)					
	(4/0.5)	(4/1)	(8/0.5)	(8/1)	(16/0.5)	(16/1)
**Strategies with gentamicin total accumulation of 240 mg**						
240 mg GEN + 1 g AZM	95.6	85.9	86.9	61.1	78.5	39.7
240 mg GEN + 2 g AZM	99.7	95.6	98.8	86.9	97.7	78.5
80 mg GEN × 3, 8 h apart + 1 g AZM single dose	95.6	86.0	86.9	61.1	78.5	39.8
120 mg GEN × 2, 8 h apart + 1 g AZM single dose	95.6	86.2	86.9	61.9	78.8	39.8
**Strategies with gentamicin total accumulation of 480 mg**						
120 mg GEN × 2, 12 h apart for 2 days + 1 g AZM single dose	99.8	99.3	97.9	93.7	92.5	76.2
240 mg GEN × 2, 24 h apart + 1 g AZM single dose	99.8	99.2	97.8	93.1	92.1	74.8
**Strategies with gentamicin total accumulation of 720 mg**						
240 mg GEN × 3, 24 h apart + 1 g AZM single dose	99.9	99.7	98.8	96.4	94.5	82.2
240 mg GEN × 3, 24 h apart + 2 g AZM single dose	100.0	99.9	99.9	98.9	99.7	95.5
240 mg GEN × 3, 24 h apart + 1 g AZM × 2, 24 h apart	100.0	99.9	99.8	98.5	99.4	93.5

Treatment is initiated at the peak NG load as identified in our model of untreated infection (at 3.6 days post-infection in the base case) (Jayasundara et al., 2019), at which point we assume symptoms to be apparent. We classify simulations in which infection is cleared in ≤7 days as treatment success, as used in recent clinical trials (Ayinde & Ross, 2020; Scangarella-Oman et al., 2018; Sultan et al., 2020) indicating this timeframe as appropriate to bound successful infection clearance. Simulated infections are assumed to be cleared when the total bacterial load ($B+B_{a}+B_{i}+B_{s}$) falls below 10 bacteria, as used in Jayasundara et al. (2019).

Any regimen that is approved for the treatment of gonorrhoea should have ≥95 % treatment efficacy (World Health Organization, 2003; Moran & Levine, 1995). Here, we adopt an analogous definition in terms of our simulations whereby for a given *zMIC* value if ≥ 95 % of simulations that are generated from our LHS samples achieve treatment success we consider that particular treatment strategy to be effective. We henceforth define simulated ‘treatment effectiveness’ as the proportion of model simulations that result in successful infection clearance. We note that the sources of variation present in our model are not directly comparable to the variability observed during the treatment of natural human infection and these percentages cannot be directly interpreted as estimates of treatment effectiveness.

### 2.5 Extracellular vs intracellular susceptibility breakpoints

To understand potential differences between *in vitro* and *in vivo* clearance behaviour, we compare the susceptibility breakpoints derived from sub-models of increasing complexity starting with only extracellular states and progressing to the full model involving epithelial cells and PMN.

Model A reflects an *in vitro* time-kill study, in which extracellular NG but no host cells (epithelial cells or PMN) are present. In simulations, NG are allowed to grow exponentially and the drug concentration is kept constant (no drug decay), similar to the experimental design used in the *in vitro* study by Foerster et al. (2016). In Model B, epithelial cells are added, leading to the inclusion of unattached NG, NG attached to epithelial cells and NG internalised within epithelial cells. In model C, NG interaction with epithelial cells is removed but the PMN response and NG survival within PMN are included in the simulations. In models B and C and the full-treatment model, logistic constraints on growth are applied as described previously in Jayasundara et al. (2019) and the drug concentration varies over time as described above in Section ‘Mathematical model of antibiotic treatment’. Comparisons of the derived susceptibility breakpoints are then made between the sub-models and the full model for the same initial extracellular drug concentration.

### 2.6 PK indices

To compare the effectiveness of differing gepotidacin treatment regimens, we evaluate three PK indices: time above the *zMIC* ($t_{\text{MIC}}$); the ratio of area under the drug concentration curve to the *zMIC* (AUC/MIC); and the ratio of peak drug concentration to the *zMIC* $\left(C_{\max}/\text{MIC}\right)$. The area integrated over the total drug concentration curve (${\text{AUC}}_{0-\infty }$/MIC) is used as the default AUC/MIC index but we also test the area under the curve above the MIC (removing the area below the *zMIC* from the total area under the curve) and AUC over a fixed time period of 7 days (${\text{AUC}}_{0-7}$/MIC) as alternative indices (see Appendix S1, Section S7.3). For multiple dose strategies, we also calculate the total time the drug concentration remains above the *zMIC* ($t_{\text{MIC}}$) and this is used as the default index of $t_{\text{MIC}}$, and additionally consider some alternative definitions of $t_{\text{MIC}}$ in Appendix S1, Section S7.3. We calculate the three PK indices separately for intracellular and extracellular drug concentrations labelling these indices with the subscripts ‘in’ and ‘ex’ (e.g., $t_{{\text{MIC}}_{\text{in}}}$; $t_{{\text{MIC}}_{\text{ex}}}$).

Similarly, for the dual treatment option we calculate the ratio of area under the drug concentration curve to the *zMIC* ($\text{AUC}/{\text{MIC}}_{h}$) using the simulated single drug concentration representing the combined effect of gentamicin and azithromycin calculated using the Loewe additivity concept (using Appendix S1, Equation S(4)). Loewe additivity combines both antibiotics, gentamicin and azithromycin into a single drug of higher effect. Here, ${\text{MIC}}_{h}$ refers to the *zMIC* of the drug having the higher effectiveness out of gentamicin (4 mg/L) and azithromycin (1 mg/L) at each time point. This PK index is calculated in both the extracellular and intracellular environments and a threshold is determined to distinguish treatment success and failure.

### 2.7 Non-adherence to treatment strategies

For multiple dose strategies of gentamicin which extend over 3 days, we also test the impact of limited non-adherence by the patient. This limited form of non-adherence reflects the fact that as gentamicin is administered intramuscularly in supervised clinical settings, extensive non-adherence scenarios such as sub-optimal doses and consecutive delays are uncommon (Brown et al., 2010). Specifically, we consider a uniformly distributed delay of between 0 and 24 h to the 2nd dose in comparison to the recommended schedule, with subsequent doses then taken at the correct spacing from the previous dose. Treatment efficacy is analysed when 15 %, 25 % 50 %, 75 % and 100 % of the simulations deriving from the LHS samples are assumed to be subject to non-adherence.

## 3 Results

### 3.1 Extracellular vs intracellular susceptibility breakpoint

For each of the sub-models described in Section ‘Simulated treatment strategies’ we determine drug-specific model-derived susceptibility breakpoints, with simulation results based on point estimates summarised together with those from the full treatment model in Table 2. In addition, breakpoint ranges derived from simulations using all LHS parameters are provided for the full model and compared with empirical breakpoints where available.

We observe that with the addition of intracellular compartments the model-derived susceptibility breakpoints are 8-fold, 14-fold and 4-fold lower in the full model as compared to the *in-vitro* model (model A) for azithromycin, gentamicin and gepotidacin respectively. Results for models B (unattached and attached NG and NG within epithelial cells) and C (unattached NG and NG within PMN) are similar to those for the full model, indicating that these large differences in model-derived susceptibility breakpoints for model A compared with the other models is associated with the inclusion of intracellular NG states in simulations.

### 3.2 Gepotidacin monotreatment

The results of model simulations for gepotidacin monotreatment are summarised in Table 3. Gepotidacin regimens that accumulate to 1500 mg in total, irrespective of administration as single or multiple doses, achieve treatment success for NG *zMIC* ≤0.5 mg/L, while most regimens with a total dose of 3000 mg achieve success for *zMIC* ≤1 mg/L. In our model, clearance behaviour is invariant when the *zMIC*/dose ratio is held fixed (see Appendix S1, Section S7.1), with higher dose strategies of 4.5 g and 6 g gepotidacin being successful for *zMIC* ≤1.5 mg/L and *zMIC* ≤2 mg/L, respectively (Appendix S1, Table S4).

Some of the multiple dose regimens for gepotidacin we investigate have not yet been tested in clinical trials. In the majority of simulated regimens, treatment success/failure is consistent across single and multiple dose strategies with the same total dose amount for the same NG *zMIC* parameter. However, daily administration of 500 mg for 6 days at *zMIC* = 1 mg/L, resulted in treatment failure (∼66 % of simulations cleared), despite treatment success with other 3000 mg total dose regimens simulated here. We discuss this result in more detail in the next section.

#### 3.2.1 Effectiveness of different dosing strategies of gepotidacin

**Fig. 2:**
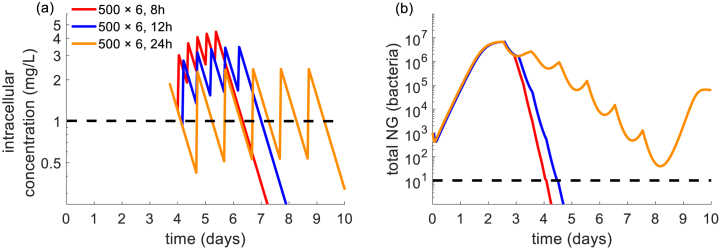
Effect of gepotidacin dosing intervals of 8,12 and 24 h in a 500 mg × 6 schedule on (a) intracellular drug concentration and (b) total NG load. Dashed lines indicate *zMIC* of 1 mg/L (a) and infection clearance cut-off of 10 bacteria (b). Parameter values are specified in Table 1.

Comparison of PK indices across the gepotidacin regimens provides insight into why the simulated 500 mg × 6, at 24 h interval regimen failed treatment at *zMIC* = 1 mg/L whereas other regimens with the same total drug did not. In this regimen, the intracellular drug concentration was maintained above the *zMIC* ($t_{{\text{MIC}}_{\text{in}}}$) for only 47 % of the dosing interval and correspondingly bacterial load spiked as the drug concentration fell below the *zMIC* (Fig. 2). By comparison, for 500 mg × 6 dosing regimens at intervals of 8 and 12 h the intracellular drug concentration is above 1 mg/L for 100 % and 94 % of the dosing interval, respectively.

The simulation results in Table 3 also suggest that in most cases, multiple dose regimens clear infection in a higher fraction of simulations when the total dose is held fixed. For instance, at a *zMIC* for gepotidacin of 0.5 mg/L infection clearance occurs in 95.0 % of simulations with a 1500 mg single dose compared with >98 % simulations in 500 mg × 3 regimens at 8, 12 and 24 h intervals. Here, the multiple dose strategies achieve an increased $t_{{\text{MIC}}_{\text{in}}}$ in comparison to the single dose strategy (Appendix S1, Fig. S9). The highest value of this PK index also occurs with the most effective dosing interval (24 h) at *zMIC* of 1 mg/L with a total dose of 3000 mg split into two (1500 mg × 2 given 8, 12 or 24 h apart) as shown in Appendix S1, Fig. S9.

#### 3.2.2 PK indices to differentiate treatment success using gepotidacin

**Fig. 3:**
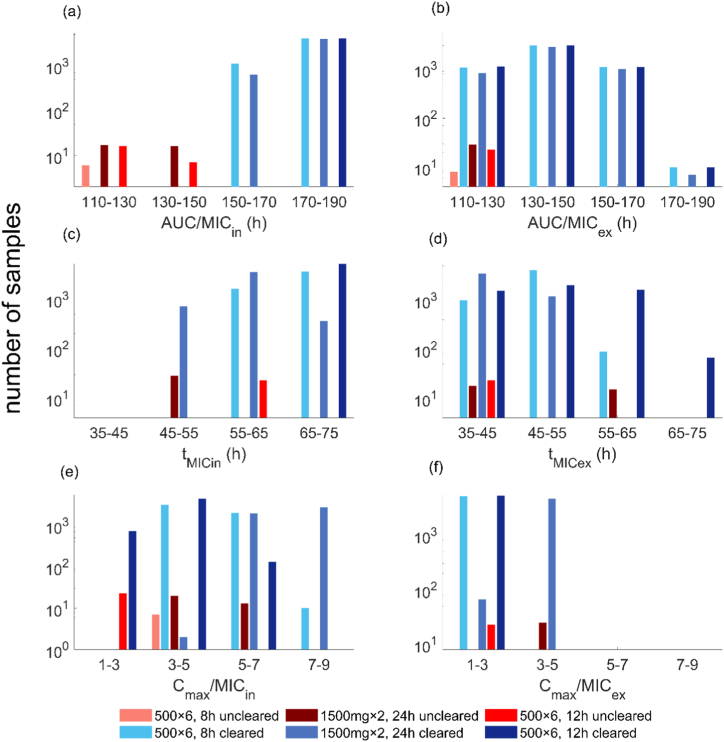
Comparison of PK/PD indices to differentiate treatment success and failure. The ratio of area under the curve to the *zMIC* are shown for: (a) intracellular and (b) extracellular drug concentration; the time above the *zMIC* calculated for intracellular (c) and extracellular (d) drug concentration; the ratio of peak drug concentration to the *zMIC* for intracellular (e) and extracellular (f) drug concentration.

We also attempt to determine treatment success and failure based on PK indices evaluated using extracellular and intracellular gepotidacin concentration. Extracellular PK indices fail to sharply distinguish simulations in which treatment succeeds from those where it fails, as there are simulations with the same PK index value but opposite treatment outcomes (Fig. 3). The ratio of peak intracellular drug concentration to *zMIC* $\left(C_{\max}/{\text{MIC}}_{\text{in}}\right)$ index is also unable to discriminate between success or failure to clear infection. In contrast, intracellular indices for the ratio of area under the total drug concentration curve to the *zMIC* ($\text{AUC}/{\text{MIC}}_{\text{in}}$) and time above the *zMIC* $\left(t_{{\text{MIC}}_{\text{in}}}\right)$, clearly differentiate between treatment success and failure. However, while a common cut-off across all dosing schedules could be obtained with the $\text{AUC}/{\text{MIC}}_{\text{in}}$ index (Fig. 3), the $t_{{\text{MIC}}_{\text{in}}}$ cut-off varies by dosing schedule. This behaviour is preserved under the alternative definition whereby only the AUC above the *zMIC* is considered (Appendix S1, Fig. S11). Dose-dependence also occurs for other forms of the $t_{{\text{MIC}}_{\text{in}}}$ cut-off (Appendix S1, Fig. S10). We therefore focus on the $\text{AUC}/{\text{MIC}}_{\text{in}}$ index for gepotidacin in regard to determination of a threshold parameter.

From the simulated concentration profiles, we observe that treatment success for gepotidacin occurs in simulations where $\text{AUC}/{\text{MIC}}_{\text{in}}$ >150 h (Fig. 3). We note that there are 6 simulations with $\text{AUC}/{\text{MIC}}_{\text{in}}$ in the range of 147–150 h that fail to clear the infection (simulation behaviour shown in Appendix S1, Fig. S12). For these unsuccessful simulations, the total bacterial load declines very close to the infection clearance threshold (to ∼11 bacteria in some instances), but does not meet our criterion for infection clearance (total NG load <10 bacteria). This further supports the $\text{AUC}/{\text{MIC}}_{\text{in}}$ >150 h, as a suitable threshold to differentiate between simulated treatment success and failure.

### 3.3 Dual treatment with gentamicin + azithromycin

#### 3.3.1 Effectiveness of different dosing strategies of gentamicin + azithromycin

The effectiveness of dual treatment with gentamicin + azithromycin across single and multiple dose strategies is summarised in Table 4. For the same total dose amount, multiple doses of gentamicin and multiple doses of azithromycin result in similar effectiveness to the single dose strategy, with limited sensitivity to dosing frequency as well. Among the tested strategies, only 240 mg × 3 gentamicin, given 24 h apart in combination with 2 g single dose of azithromycin is effective at high *zMIC* for both gentamicin and azithromycin (16 mg/L and 1 mg/L, respectively, Table 4). We also examine the impact of limited non-adherence using the multiple dose strategy of 240 mg × 3 gentamicin, given 24 h apart along with 2 g single dose of azithromycin. Here, at *zMIC* for gentamicin and azithromycin of 16 mg/L and 1 mg/L, respectively, for the 100 % non-adherence scenario 94.13 % (Appendix S1, Table S5) treatment success is observed showing similar effectiveness (95.45 %) to the 100 % adherent scenario (Table 4).

#### 3.3.2 PK index to differentiate treatment success using the dual treatment combination of gentamicin and azithromycin

**Fig. 4:**
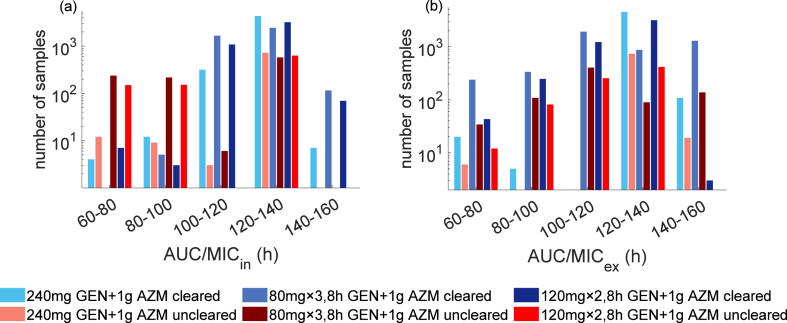
Simulated infection clearance based on the ratio of area under the (a) intracellular and (b) extracellular drug concentration resulting from Loewe additivity $\left(\text{AUC}/{\text{MIC}}_{h}\right)$ for gentamicin and azithromycin dual treatment option.

We also attempt to distinguish treatment success and failure based on the PK index evaluated using the single drug resulting from Loewe additivity. Similar to gepotidacin, the ratio of area under the total intracellular drug concentration curve to the *zMIC* ($\text{AUC}/{\text{MIC}}_{h,\text{in}}$) can clearly differentiate between treatment success and failure (Fig. 4). From the simulated concentration profile of a single drug resulting from Loewe additivity, we observe that all samples achieving $\text{AUC}/{\text{MIC}}_{h,\text{in}}$ > 140 h successfully clear infection. However, unlike the PK index threshold related to gepotidacin monotreatment, we observe that a substantial proportion of simulations successfully clear infection when $\text{AUC}/{\text{MIC}}_{h,\text{in}}$ < 140 h.

## 4 Discussion

In this study, we develop a within-host mathematical model to describe antibiotic treatment effects while considering NG interactions with host cells. We found that inclusion of intracellular states leads to substantial changes in *zMIC* clearance thresholds as opposed to *in-vitro* NG dynamics alone. The relevance of different intracellular NG states in determining treatment success is a matter of current debate by experts in this field (Theuretzbacher et al., 2020). The difficulty in reaching a consensus on this issue is likely due to limited experimental evidence of the impact of intracellular antibiotic-mediated killing on treatment outcomes. Here, our findings on the model-derived susceptibility breakpoints and treatment effects in the presence of intracellular NG, suggest further experiments assessing the role of intracellular NG in determining treatment success could be valuable. We also analyse the association of PK indices with treatment success and the level of intracellular drug concentration that must be maintained to achieve successful infection clearance. When calculating PK indices relevant to the dual treatment option we introduce a novel approach of using a simulated single drug concentration representing the combined effect of gentamicin and azithromycin calculated using the Loewe additivity concept. However, unlike in the monotreatment case, the threshold relating to dual treatment does not separate treatment success from treatment failure as a majority of samples below our PK index threshold still lead to clearance.

In Jayasundara et al. (2019), we showed the importance of intracellular NG in prolonging the duration of natural infection and here we show the importance of intracellular antibiotic mediated killing in determining treatment success in our model. The importance of different intracellular NG states (NG within PMN and epithelial cells) in determining treatment success is not as yet resolved (Theuretzbacher et al., 2020), due to limited experimental evidence of the impact of intracellular antibiotic-mediated killing on treatment outcomes. Although *in vitro* models such as those developed using immortal cell lines (e.g., HeLa cells) (Gubish et al., 1979) have been used to explore the intracellular behaviour of NG, we are not aware of any study that considers antibiotic interactions with intracellular NG. Here, our findings on the model-derived susceptibility breakpoints in the presence of intracellular NG, suggest further experiments assessing the role of intracellular NG in determining treatment success could be valuable.

Building on our simulation results highlighting the importance of intracellular concentrations in treatment success, we found that an intracellular version of the area under the curve index discriminated between treatment success and failure using gepotidacin. Consistent with our findings, a strong correlation between AUC/MIC index and bacterial killing of two gram-positive pathogens (*S.aures* and *S. pneumoniae*) has been reported by Bulik et al. (2017). Although our extracellular index measures align with the calculations based on plasma drug concentrations in Scangarella-Oman et al. (2018), their study is limited by sample size with only five NG isolates with MIC for gepotidacin of 1 mg/L (Scangarella-Oman et al., 2023). However, in our model treatment success and failure could only be clearly differentiated through intracellular indices. This is because, in our model implementation, consistent with limited empirical evidence of intracellular NG populations measured in urethral exudates by Veale et al. (1979), a majority of NG reside intracellularly (Jayasundara et al., 2019) and here, treatment success is observed to be mainly determined through the killing of intracellular NG (Table 2).

Our analysis of dual treatment using single doses of gentamicin + azithromycin is comparable, to a certain extent, with the limited data available from clinical trials. The two clinical trials that have been conducted for this drug combination report an overall genital infection treatment success rate of 94 % (Ross et al., 2019) and 100 % (Kirkcaldy et al., 2014) using 240 mg gentamicin combined with 1 g and 2 g azithromycin doses, respectively. In the clinical trial by Ross et al. (2019), 97.7 % and 95.7 % of isolates had MIC for gentamicin ≤4 mg/L and MIC for azithromycin ≤0.5 mg/L, respectively. However, in these studies treatment success is not disaggregated into MIC ranges and therefore, a clear comparison cannot be made with our model simulation results for *zMIC* for gentamicin and azithromycin of 4 mg/L and 0.5 mg/L, respectively. The gentamicin susceptibility breakpoint of 4 mg/L was based on a single study from Malawi (Brown et al., 2010), given the lack of other clinically validated estimates in the literature. Given known heterogeneity amongst NG strains observed internationally (Mann et al., 2018), this breakpoint should be viewed with some caution in terms of extrapolating our results to differing settings.

In real-world sexual transmission networks, most circulating NG isolates have experienced prior antibiotic exposure, and therefore their susceptibility profiles reflect accumulated selective pressure rather than wild-type behaviour. However, given the absence of time-kill data on such isolates, we adopted a model-based approach to reduced susceptibility, first fitting to *in-vitro* time-kill experiments on fully susceptible strains (Farrell et al., 2017; Foerster et al., 2016) and then changing the *zMIC* parameter to capture decreased susceptibility in simulations. This approach has the advantage of simplicity but lacks a mechanistic formulation of the development of resistance, so that the model is unsuitable at present for studies of the development of resistance.

Furthermore, our model does not explicitly consider host factors such as co-morbidities or mixed infection, which are known to alter treatment outcomes (Terreni et al., 2021; Unemo et al., 2016). As such, results should be interpreted as applying to single-strain uncomplicated infection, with caution applied in terms of extrapolation beyond this.

Further, while gepotidacin has shown promise in recent phase 3 clinical trials, it is not yet widely accessible, particularly in many LMIC settings, where the burden of gonorrhoea is disproportionately high (Kirkcaldy et al., 2019). While modelling gepotidacin treatment dynamics provides insight into the PK/PD requirements for successful treatment, real-world implementation would depend on factors such as future regulatory approval and availability within public health systems. Therefore, the applicability of our gepotidacin-related findings to global clinical practice remains contingent on future expansion of access.

If additional data on antibiotic mediated killing of intracellular NG become available through future experimental studies, analogous for example to the *in vitro* time-kill experiment by Barcia-Macay et al. (2006) that analysed drug mediated killing of extracellular and intracellular *S. aureus,* some of our findings on the rates of intracellular NG killing by antibiotics could then be compared with experimental data. Although such experimental studies on antibiotic activity against other intracellular pathogens can be a useful guide it is important to note that the magnitude of intracellular bacteriostatic/bactericidal effects depends on both the pathogen and the drug (Van Bambeke et al., 2006).

While most PK parameters (e.g., volume of distribution, drug half-life) are based on plasma drug concentration profiles measured in patients, we have had to rely on *in vitro* data for the PD parameters and some PK parameters. The experimental limitations of these *in vitro* studies, such as the use of constant drug concentrations and lack of intracellular bacteria, do not reflect the true *in vivo* environment and add potential for error in these parameters. In addition, the studies by Ripa et al. (1996) and Schentag et al. (1977), used to inform intracellular drug accumulation have some differences in terms of compartment and cell types to those modelled in our study. Ripa et al. (1996) study drug concentrations in plasma and extravascular compartments, while Schentag et al. (1977) measured drug accumulation in tissue but not in PMN or epithelial cells. As drug accumulation can vary depending on the type of cell (Kong et al., 2017; Van Bambeke et al., 2006), this imprecision is a limitation of our study. Reflecting the limited data available, we took a parsimonious approach in assuming that intracellular PK effects for PMN and epithelial cells were the same. Although we recognise that both drug accumulation and penetration can depend on the host cell and tissue type (Kong et al., 2017; Van Bambeke et al., 2006) we lacked relevant data to inform different estimates. To mitigate these limitations to a certain extent, we used LHS sampling where these PK parameters were sampled over a broad range (Table 1). However, when assessing treatment success rates based on simulations from LHS samples, the sources of variation in our model do not directly correspond to the variability observed in real-world treatment of human infections. As a result, the reported success rates cannot be directly interpreted as estimates of actual treatment effectiveness.

## 5 Conclusions

In this study, we developed a PK/PD analysis approach to study antibiotic interaction with NG in different cellular states and to assess the effectiveness of novel treatment strategies over a range of MIC values. To the best of our knowledge, this is the first within-host mathematical modelling study that explores the intracellular antibiotic killing of NG. Our findings suggest the importance of considering intracellular dynamics when deciding on treatment regimens as the model-derived susceptibility breakpoints are observed to be substantially impacted by the killing of NG within PMN and epithelial cells. This also draws attention to the potential importance of further experimental studies that capture intracellular PK/PD effects in regard to gonorrhoea treatment. Such investigation into the intracellular antibiotic effects may be useful when developing novel antibiotics for gonorrhoea. In addition, our findings, and the model more generally, may have utility as a tool for identifying treatment regimens to explore further in clinical trials.

## CRediT authorship contribution statement

**Pavithra Jayasundara:** Writing – original draft, Visualization, Methodology, Formal analysis, Conceptualization. **David G. Regan:** Writing – review & editing, Supervision, Methodology. **Philip Kuchel:** Writing – review & editing, Supervision, Methodology. **James G. Wood:** Writing – review & editing, Supervision, Methodology.

## Funding

Author's declare no competing interests. This work was supported by funding from the National Health and Medical Research Council (NHMRC) [grant numbers APP1078068 and APP1071269]. PJ was supported by a UNSW Sydney PhD tuition fee scholarship.

## Data availability

All relevant data are within the paper and its Supporting Information files and the code is available on GitHub at https://github.com/pavijayasundara/NG-Treatment-Model.

## Declaration of competing interests

Author's declare no competing interests.
